# Spectrally selective antireflection of nanoimprint lithography-formed 3D spherical structures on film coated with a silver layer

**DOI:** 10.1038/s41598-022-23348-w

**Published:** 2022-11-14

**Authors:** A. H. Chiou, C. W. Chang, C. J. Ting

**Affiliations:** 1 Department of Mechanical and Computer-Aided Engineering, National Formosa University, Yunlin, 632 Taiwan, ROC; 2 Department of Optimal Pattern Correction Technology, Taiwan Semiconductor Manufacturing Company, Hsinchu, 300 Taiwan, ROC; 3 Mechanical and Systems Research Laboratories, Industrial Technology Research Institute, Hsinchu, 300 Taiwan, ROC

**Keywords:** Nanoscale materials, Composites

## Abstract

We fabricate moth-eye antireflection (AR) coatings using high-resolution and low-cost UV nanoimprint lithography with polyethylene terphthalate (PET) molds. Several various thicknesses of silver films placed on the moth-eye structure were analyzed for reflectance and transmission. On PET, the conical nanostructured surface arrays had a spatial period length of approximately 250 nm, a diameter of approximately 200 nm, and a height of approximately 160 nm. After them, a silver (Ag) layer of 18 nm is deposited satisfactorily on the PET substrate surface. The never-ending moth-eye formations of the imprinted mold were fabricated by Ni mold electroplating, interference lithography, and replication. We found that an Ag layer of suitable thickness on AR film in the spectrum range that can be seen has high transmittance (Highest value is 72%) while in the infrared spectrum it has high reflectance (At least 60%). For an optical film with a silver coating has been placed on an anti-reflection subwavelength-structured (ASS) surface, such properties, including heat insulation, have obvious applications in windows for homes and vehicles.

## Introduction

With the improvement of technology, the demand for entertainment by human beings becomes higher and higher, therefore, the characteristics of light obviously become the important topic. To keep unwanted reflections at bay, glare as well as ghost images in digital screens, solar panels, lighting and optics, vehicle thermal insulation, storefronts, architectural glazing and others is very important. Anti-reflective (AR) coatings are one approach that is now being used to eliminate exterior reflections. An anti-reflection coatings have been researched widely because they play a vital role in architectural glasses^1^, solar photovoltaic devices^2,3^, laser systems^4^, light emitting diodes (LED)^5^, display screens^6^ and others^7^. At present, two approaches to reducing the reflectivity are used. The first method, similar to thin-film technology, includes alternating layers of high and low refractive index^8^. The second, found in antireflection structure technology ^9–11^, is an inhomogeneous film with a gradual change of index^9–11^. The multilayered modification of high and low refractive index layers separates an incident beam into a reflected beam and a transmitted beam as optical rays move through the surface of a film in coated multilayer films. The reflecting wave destructively interferes with the incident wave if the optical film thickness (the refractive index multiplied by the film thickness) is an odd multiple of one-fourth of the wavelength of the incident light. Eventually, at a given expected position, the reflection approximates to zero. With a wide waveband, to reach an extremely low reflection, the best current solution is a film with multiple layers and a complex structure that is antireflective in a specific waveband. While thin-film technology is mature, there are still limitations in the coating materials, as well as issues with the physical and chemical properties of the thin film stack that affect thermal mismatch, adhesion, and stability. Thus, anti-reflective structural surfaces (ARS) are proposed as an alternative^12–14^. The technology involves layering the element surface and sub-wavelength structure for anti-reflection to form an anti-glare sub wavelength structure (ASS) surface.

Recently, researchers have to fabricate efficient AR materials with 3D nanostructure AR films^15–17^ to get better anti reflection. Among them, compared with the traditional multilayer antireflection coatings, a moth-eye nanostructure inspired by nature have the advantages of excellent wideband, little glitter and elevated transmittance of wide-angle incident light. It is considered to be a successful method to boost the visibility and photoelectric conversion efficiency of the display^2,3^ and can also effectively block most of the radiant heat^18^. Furthermore, global warming, energy depletion, climatic changes, and other environment-related issues in recent years have led to the appearance of green technologies. Therefore, green energy technology has always been a hotbed of study. There is a simple way to cut energy usage by making a building or automobile window work as a bandpass filter, with poor transmittance of sunshine in the UV and infrared spectral regions and sufficient transmittance in the visible spectral range. It is clear that the film with the aforementioned function, commonly referred to as heat insulation film and manufactured using moth-eye surfaces, gums up the glass^19^. Several methods have been developed to synthesize moth-eye AR surfaces successfully, such as electron beam lithography^20^, interference lithograph colloidal lithography^21^ or others^22^. Continuous nanoimprint printing, as described above, may be the most ideal technology for producing moth-eye AR surfaces on an industrial scale for items such as vehicle thermal insulation sheet. It is necessary not only to reduce reflection, but also to have the advantages of transparency, mechanical strength, stability and durability. The use of nanoimprinting to create effective moth-eye AR polymer films has been reported^23^. However, various issues must be overcome before the technology can be put into practice, including the fragility of the features, the adsorption of pollutants from the atmosphere, and possibly degeneration with time, to name a few. Therefore, in order to solve the above problems, the silver film has been coated on the structure of the moth's eye to make a thermal insulation membrane with low light reflection rate and low infrared light penetration rate. Sliver film have chemical reaction in the air, has high stability, good mechanical and adhesion, therefore they have important application value. In addition, silver has low reflectivity in the region that is visible with an elevated reflectivity in the infrared spectrum higher than that of aluminum, it is suitable for plating on the thermal insulation film.

Since this research can be used in vehicle thermal insulation and green buildings in the future, the developed AR film needs high reflection in ultraviolet and infrared bands and low reflection in visible light. The traditional thermal insulation membrane mainly adopts multilayer optical film coating or metal film coating to block ultraviolet and infrared rays. When the general membrane blocks the light in the infrared and ultraviolet bands, it is easy to reduce the light penetration greatly, affecting the indoor daylighting; At the same time, due to the high reflection rate of the window surface, after the light is reflected by the metal surface, it will cause internal reflection and easy to produce obvious reflection. At present, the commercially available thermal insulation membrane can block more than 99% of ultraviolet rays and 35 ~ 97% of infrared rays. Therefore, on the premise of blocking a large amount of thermal radiation, this study intends to manufacture a moth eye structure with silver film, hoping to improve the light penetration and reduce the medial reflection. Many studies have been demonstrated various metal coating based on moth-eye structure. Supplementary Table S1 shows the comparison of parameters and reflectivity and transmission based on different metal-based moth-eye structure. It can be seen from Table S1 that the moth eye structure with metal can be formed by allowable manufacturing methods.

Throughout this study, we examine the use of a moth-eye AR surfaces coated with silver layer of differing thickness. We expect high reflection in the spectral range 850 nm to 1800 nm and transmission between 400 to 800 nm. This study presents an experimental study of the use of a silver layer in that role. These we compare with widely used commercially available samples. These commercial products do not offer low transmittance of daylight in the infrared spectral ranges as well as the ultraviolet, and relatively little transmittance in the spectral range that is visible. The thermal-insulation films fabricated in this study clearly outperform the commercial products. Our findings reveal that in the visible spectral region, an element with a silver layer producing an antireflection subwavelength structured surface has good transmittance and minimal glare, whereas in the infrared range, it has high reflectance.

## Experimental procedures

**Figure 1:**
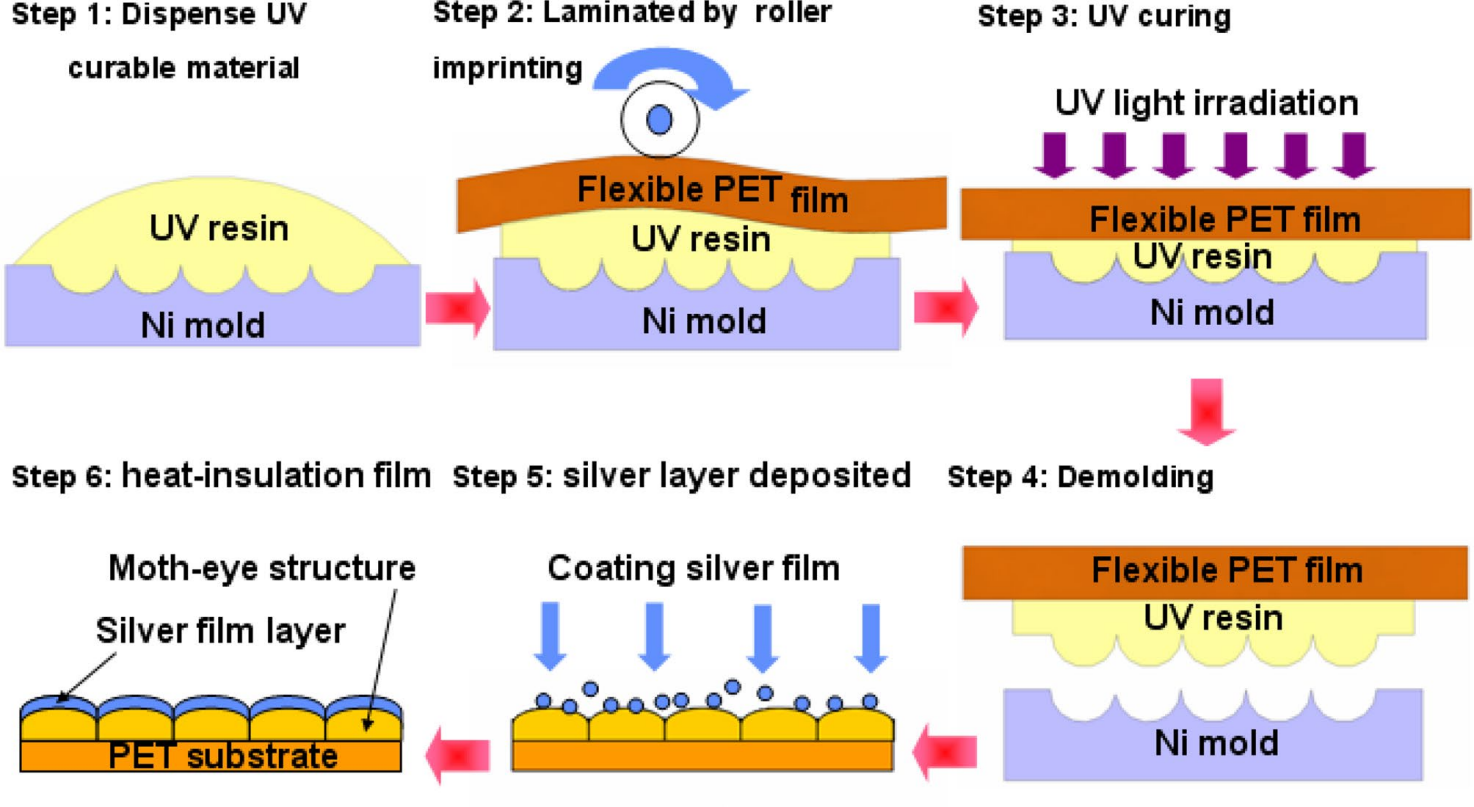
Schematic illustration of antireflection by nanoimprint lithography.

To raise the conversion efficiency, we must lower the reflection of sunlight via by using accumulations of layers with differing refractive indices to form antireflection (AR) coatings. This type of structure can alter the effective reflective index. Figure 1 depicts the fabrication steps. First, UV curable liquid material is delivered onto the Ni template in the ASS's concave cavity. To prevent air bubbles from becoming trapped in the resin layer, an ASS substrate made of flexible polyester (PET) film is placed over the dispensed resin. Toyobo PET film (thickness 185 μm) from Toyobo Co. Ltd., Osaka, Japan is used. UV light does not penetrate the PET film. The PET film-coated template is then laminated with a height-controlled roller that moves from one side to the other, removing redundant resin and controlling the UV resin layer thickness. The coated resin layer is therefore given a thickness of 200 μm. UV light with a wavelength region of 250 to 420 nm is used in the experiment. The intensity of its exposure is 40 mW/cm^2^ at a 365 nm wavelength, as given by a photometer. We then cure the sample for 12 min and solidify the resin at room temperature. Next, we remove the flexible PET film with ASSs from the Ni mold. Using an advanced plasma-enhanced magnetron sputtering (PEMS) system, we then deposit a silver layer on the subwavelength structure. For analysis of the silver layer’s optical characteristics as well as the subwavelength structure, the subwavelength structures of the silver layer on the PET substrate have thicknesses of 18, 25, 50, and 75 nm. In this research, transmittance and reflectance are measured using a model Jasco V-570 commercial spectrophotometer. The measurements of these characteristics fall into the zeroth order range.

Finally, the light transmittance differences of three kinds of thermal insulation films are discussed, including two kinds of commercial thermal insulation films (K-100SR of SunMark and AL-608 of RAL company) and the thermal insulation films manufactured in the current research. The SunMark technology was used nanoparticles conversion property via nano-grinding/dispersion technology. The RAL company produced the AL-608 that prepared by dye technology.

## Results and discussion

**Figure 2:**
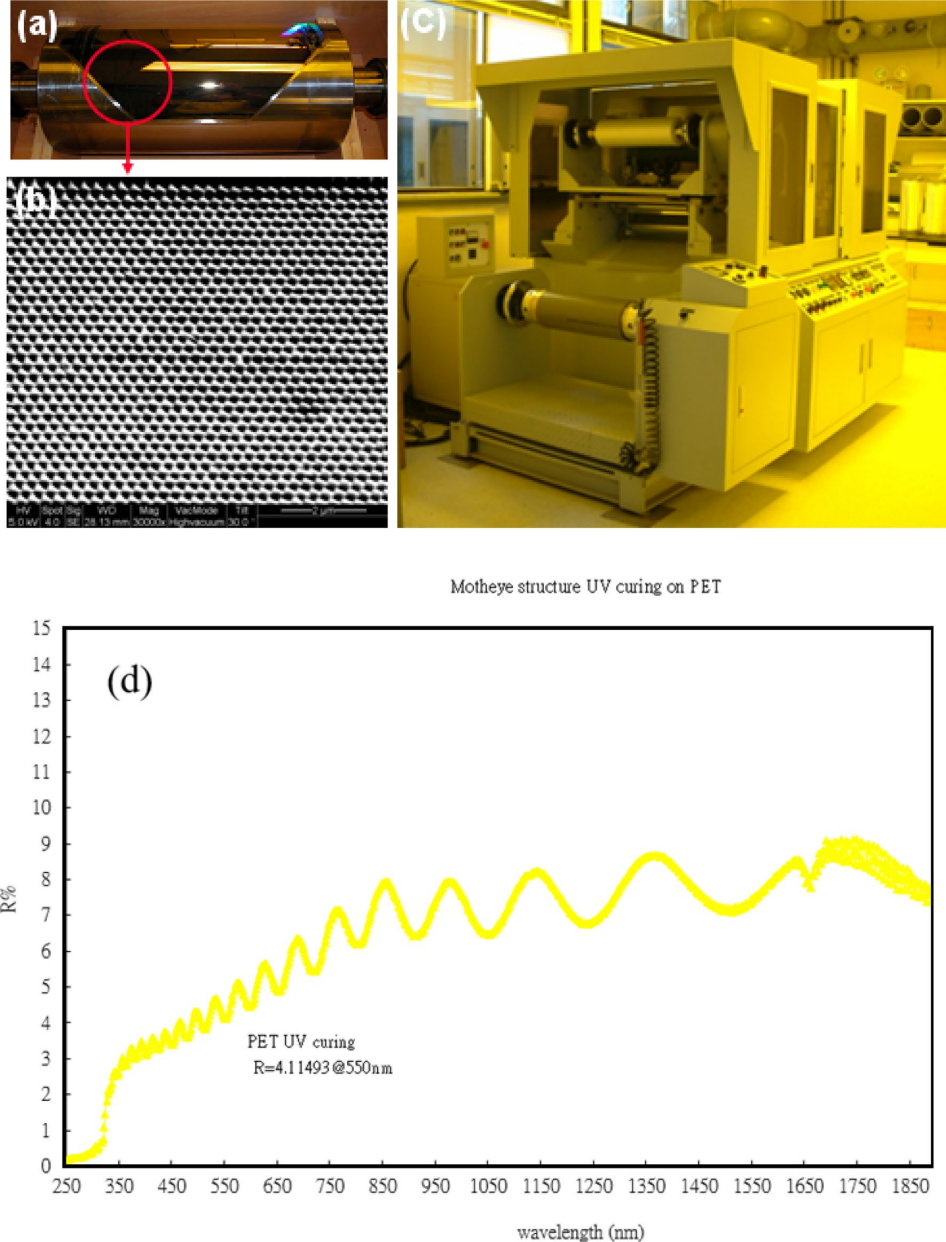
Fabrication of the moth-eye structures: (**a**) bonded roller mold adhered to the ASS-based Ni mold. (**b**) SEM image showing the cylindrical ASS surface on the Ni mold. (**c**) Image showing the roller imprinting equipment of our own design for continuous nanostructure duplication. (**d**) Variances in the transmittance of light for motheye coating without Ag.

A micro-replication process combining Ni mold electroplating, interference lithography, and replication via UV imprinting into plastics are used to fabricate the structures in this research. Interference lithography can fabricate periodic structures with sub-micro periods and interference patterns made up of at least two coherent waves superimposed on top of each other. In this experiment we use a modified imprinting process employing a roller. Figure 2a depicts the bonded nanostructure roller mold (20 cm in diameter), while Fig. 2b presents a manufactured SEM photo of the Ni mold. It is made with nanoimprinting equipment from ITRI (Fig. 2c). Figure 2d shown variances in the transmittance of light for motheye coating without Ag. The reflectance of PET with nanostructures ranges from 4.12 to 8.0% in the wavelength of 400–800 nm.

**Figure 3:**
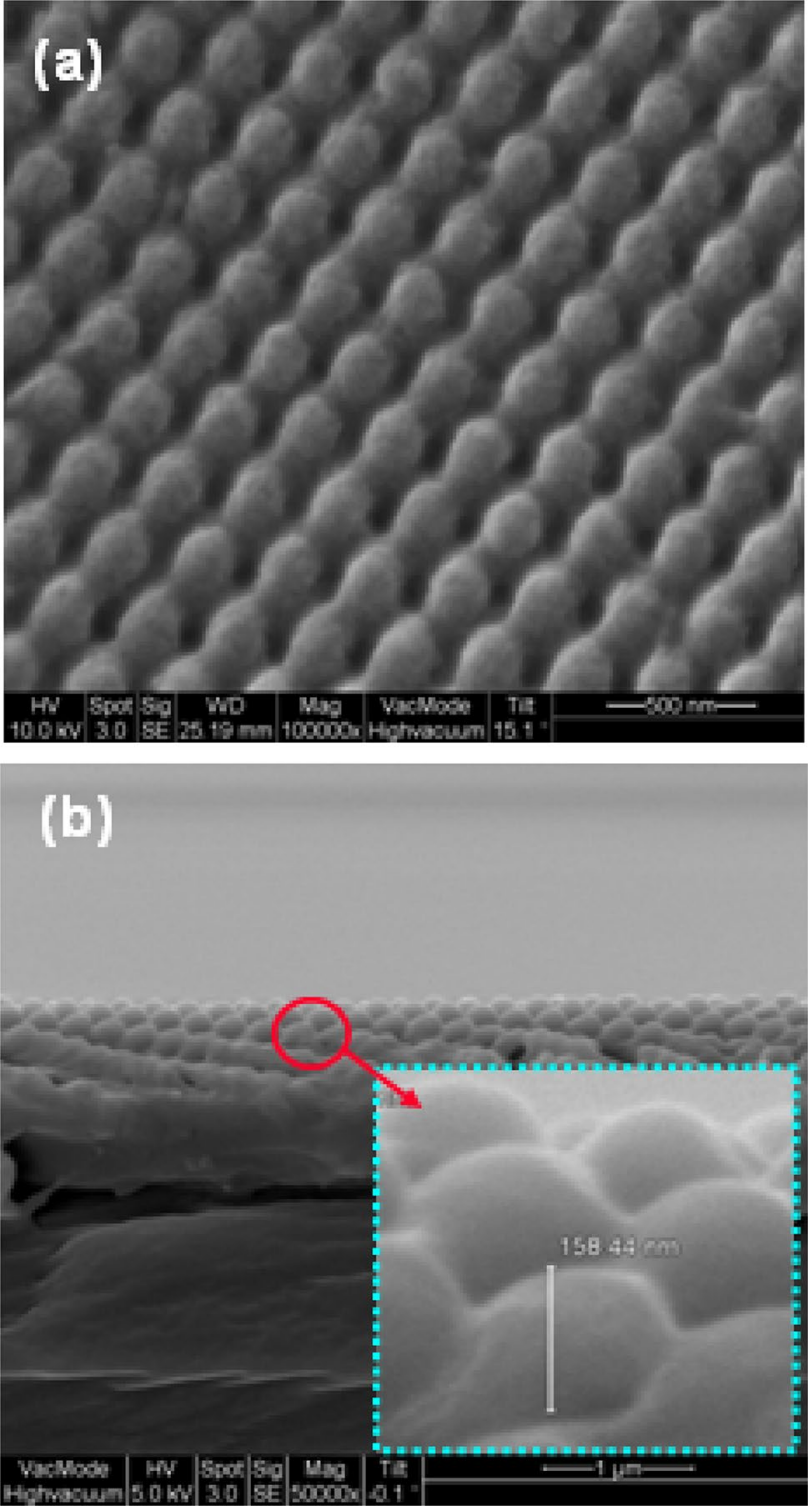
SEM photo of the conical ASS surface: (**a**) order periodic nanostructures, and (**b**) cross-sectional nanostructures.

Employing UV nanoimprint lithography onto PET substrate, the micro replication process we use comprises interference lithography, Ni mold electroplating, and replication, along with structure template fabrication. Figure 3 presents an SEM photo of the prototype, showing success sful duplication of the ASSs. The conical nanostructured surface arrays have a spatial period length of about 250 nm and a diameter of about 200 nm and height of about 160 nm on PET.

**Figure 4:**
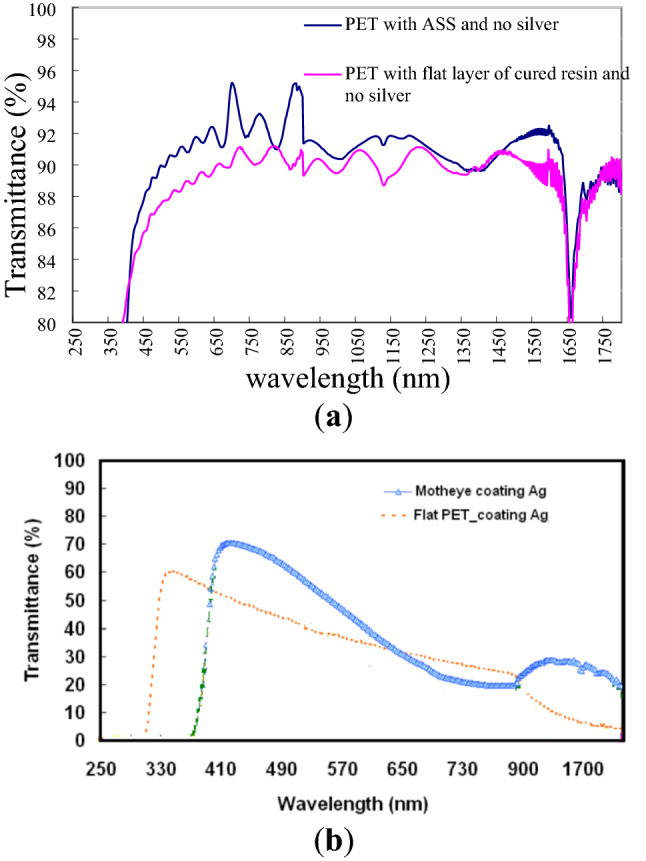
Variances of transmittance of light propagating through optical films: (**a**) flat PET and ASS without silver and (**b**) flat PET and ASS with silver coating.

Figure 4a shows the transmittance of flat PET and ASS without silver. The results show that when the substrate is moth eye structure, the transmittance is better than flat pet in the wavelength range of 450–1200, which is attributed to the only allowed zeroth-order wave at wavelengths much higher than the period of 380 nm, as well as the gradient effective refractive index profile via conical gratings, which is known as the "moth-eye effect^24^". Figure 4b shows the shows the transmittance of flat PET and ASS with same silver. The penetration rate of the moth eye structure with silver layer is superior than that of the flat pet, as shown in the figure. However, the penetration rate of moth eye structure with metal layer is lower than that without metal layer due to SPR effect^25^.

**Figure 5:**
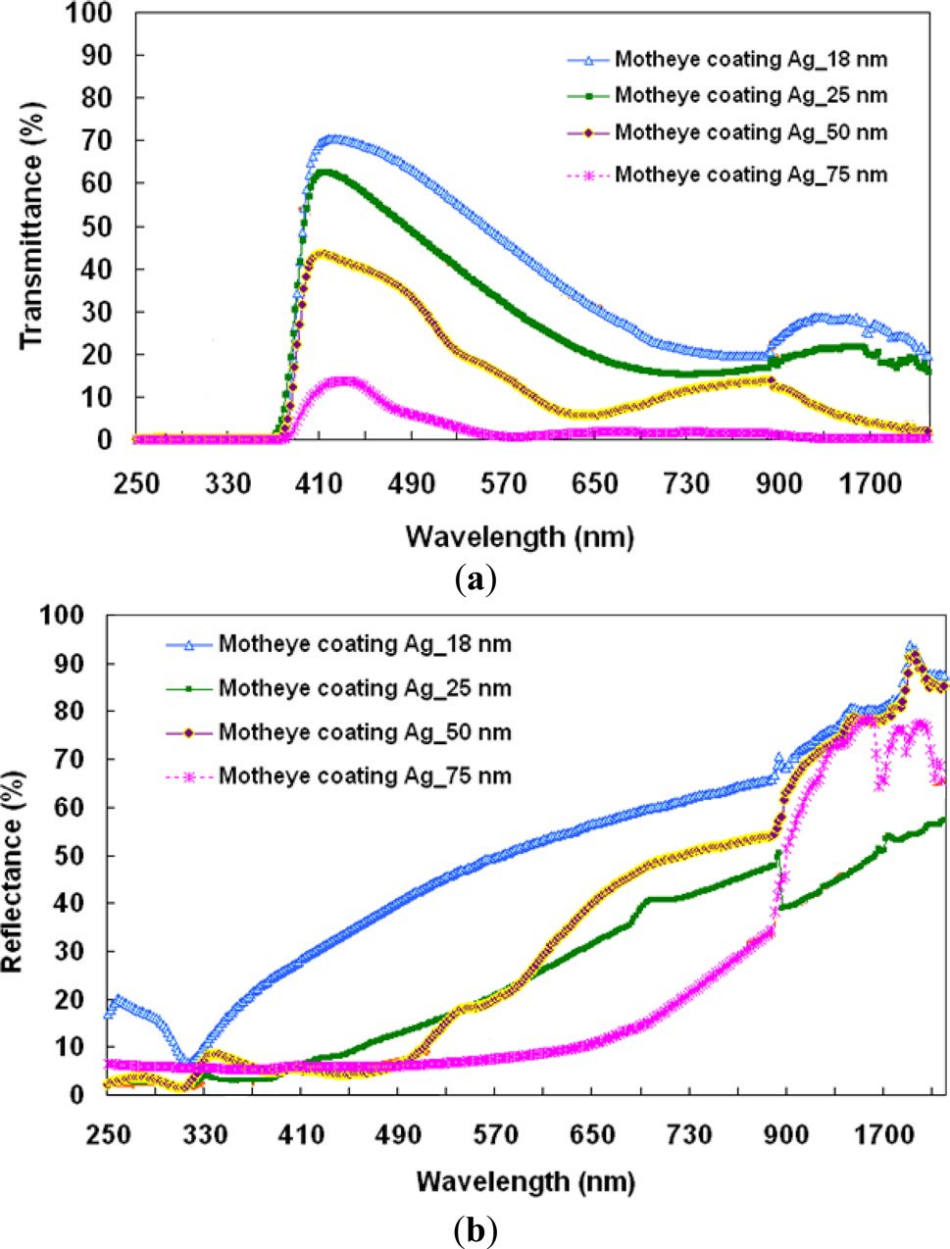
Variances of (**a**) transmittance and (**b**) reflectance of light propagating through optical films with the ASS surface coated with silver layers of different thicknesses.

Figure 5 highlights the variances in (a) transmittance and (b) reflectance of light in the optical films, including a bare ASS surface and the four ASS surfaces with silver layers of differing thicknesses. The transmittances clearly fall when the ASS surface has a silver layer. The transmittance decreases with the thickness of the Ag film, as shown in Fig. 5. The reflectance of the Ag film can also be measured, and it rises with its thickness. The transmittances remain at large values exceeding 40% in the visible wavebands, except with the silver layer of 75-nm thickness. Especially in the infrared, ASS surface reflectance increases greatly when coated with a silver layer. This property is due to the Ag-coated pillars, which effectively limit and localize light around them. That is to say when an Ag mirror layer is present, the localized surface plasmon resonance (LSPR) effects are increased, resulting in gap plasmon resonance induced by the metal–dielectric–metal nanostructure, which gives rise to gap plasmon resonance caused by the metal–dielectric–metal nanostructure. Moreover, the silver film has high reflectivity to the infrared region because there are many electrons on the surface. A thin Ag film with high transmittance in the visible waveband and strong reflection in the infrared range could be a good alternative for vehicle or building window glass as long as a continuous Ag film can be fabricated on the moth-eye structure.

**Figure 6:**
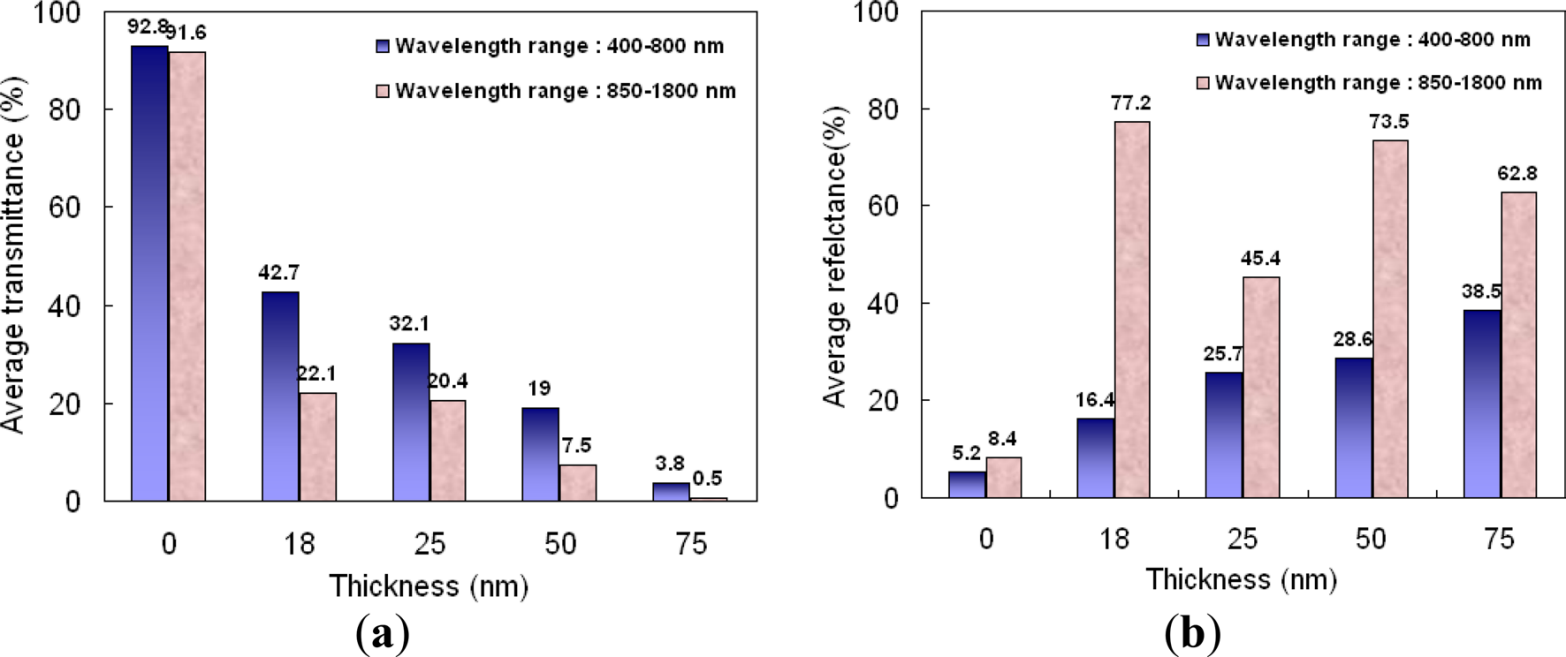
Averages of (**a**) transmittance and (**b**) reflectance of light propagating through the optical films with the ASS surface coated with silver layers of different thicknesses, and the bare ASS surface for the spectrum ranges from 400 to 800 nm and 850 to 1800 nm.

**Figure 7:**
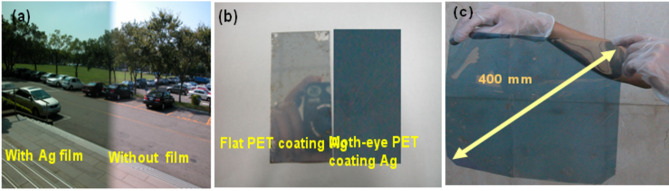
Images of antireflection: (**a**) and (**b**) images are results for comparison. Here surfaces of the film are covered with an Ag layer 18 nm thick. (**c**) A large area replicated element of the AR film.

For light in the spectrum ranging from 400 to 800 nm and from 850 to 1800 nm, (a) transmittance and (b) reflectance averages for the optical films are given in Fig. 6. For silver thickness of 75 nm, Fig. 6a shows that the average transmittance is only 3.8% in 400 to 800 nm range. With increasing metal layer thickness, the power of the transmitted light declines as the incident light passes through the metal layer. It is likely that the 75 nm silver layer is too thick, reflecting most light. The 18 nm and 25 nm layers have maximum average transmittances above 42.7% in the 400 to 800 nm spectrum range, while in the 750 to 1800 nm range the maximum average transmittances are just under 20%. In the 50 nm layer average transmittances are about 19% and 7.5% for 400 to 800 nm and 850 to 1800 nm, respectively. Maximum average reflectances are 77.2% in the 850–1800 nm range for the 18 nm silver layer. The antiglare features of optical film are strongly affected by these numbers. These results show that the metal film reduces the transmittance in the 850–1800 nm range, while the 18 nm silver film has high reflectance in that range. To illustrate the effects of these optical films, we use a digital camera whose lens is coated without optical films and with the moth-eye structure with the 18 nm silver layer to generate the images shown in Fig. 7a. Photo of the flat optical films covered with the 18 nm thick silver layer. The left has no moth-eye structure while the other has the 18 nm thick silver layer (Fig. 7b). The mirrored digital camera in the photographer's back is visible through the Ag film on the flat PET substrate. The view through the Ag film on the moth-eye structure has less reflected scenery. Besides, the moth-eye structure was prepared by continuous nano-imprinting method. The study successfully confirmed that the diagonal sample of about 400 mm can be prepared in this study. A picture of a large area sample, with a diagonal size of about 400 mm, is shown in Fig. 7c. It shows the antireflective effect of the 18 nm thick duplicated nano-structure.

**Figure 8:**
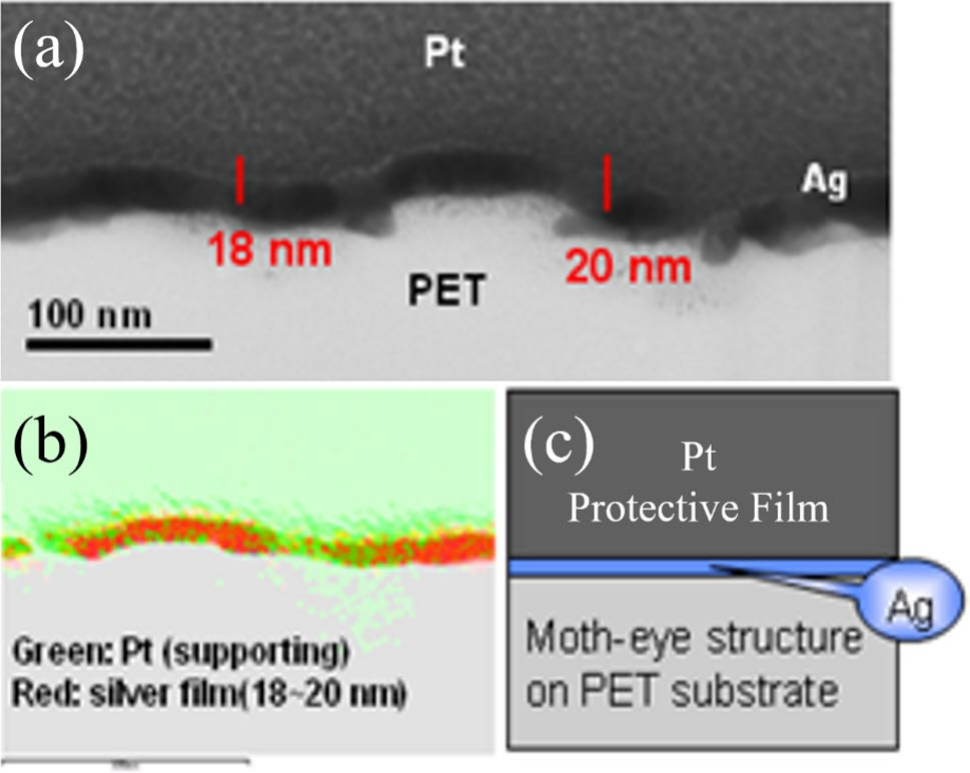
Analysis of the moth-eye structure with silver film 18 nm thick and in antireflection film with a protective layer of Pt material. (**a**) TEM images, (**b**) elemental mapping analysis and (**c**) Schemat-ic of Ag-motheye mixed structure-based ASS structure.

Figure 8 presents a transmission electron microscope (TEM) image and elemental mapping of the conical nanostructure with silver film and a protective layer of Pt material. The TEM images in Figs. 8a illustrate that Ag film and a protective layer of Pt material are deposited onto the moth eye structure of PET substrate. Ag film thick are in the range of 18–20 nm. In Fig. 8b, the Ag single in elemental mapping of the moth eye structure of PET substrate further confirm the presence Ag film on the moth-eye structure. In addition, in this study, hard Pt film was introduced as sample protective layer that to protect the surface of the sample from scratch, avoid oxidation and prolong the product life.

**Figure 9:**
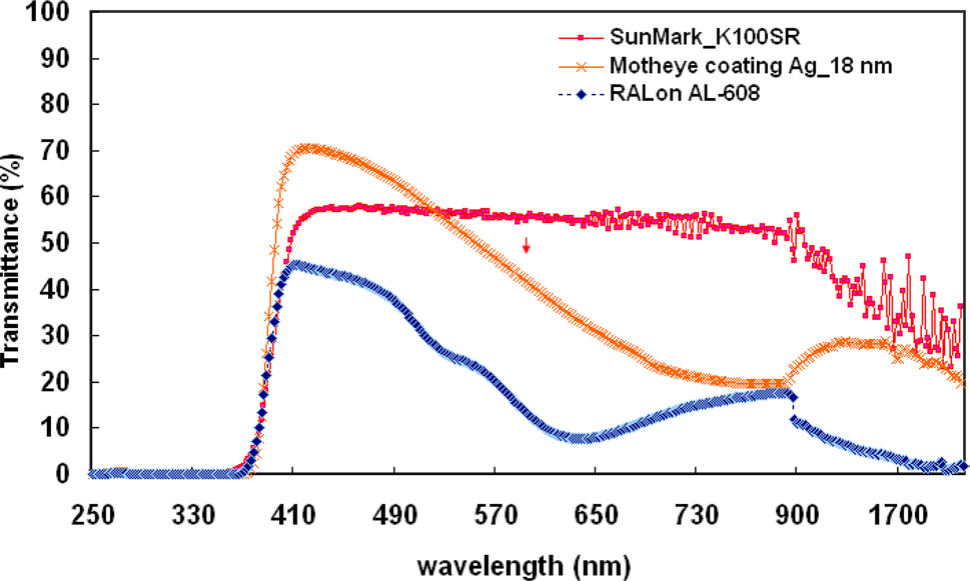
Variances in the transmittance of light for three thermal insulation films including two com-mercially available and the one fabricated in the current study.

One is the type AL-608 produced by the RALon company, the other is the K-100SR, produced by SunMark. Variances in the transmittance of light by three commercial thermal insulation films is presented in Fig. 9. For the K-100SR, the AL-608, and a moth-eye silver coating of 18 nm the average transmittances are roughly 56%, 26%, and 65% in the 400–800 nm spectrum range and nearly 42%, 13%, and 24% in the 850–1800 nm range, respectively. Although the thermal insulation film coated with metal layer can effectively block ultraviolet and infrared light, it also has the effect of shielding light. In this study, the Ag-coated moth-eye film provides a large-area broadband light absorption medium that is useful for green building, vehicle thermal insulation or optical devices. In Fig. 9, the proposed structure has low transmission at the visible wavelength of 570–800nm. Because there are many electrons on the metal surface, which will produce scattering phenomenon. Therefore, it has high reflectivity at the visible wavelength of 570–800 nm.

To sum up these experimental results, Table 1 shows the transmission and reflectance of two commercially available and the one fabricated in the current study are summarized in different wavelength. Compared with al-608, the moth eye structure with silver film has higher transmittance in the visible light range. Then, in the 850–1800 band, the transmittance of moth eye structure with silver film is lower than k-100sr. This is due to the SPR effect caused by more electrons on the surface when the moth eye structure is covered with a metal film^25^. Nevertheless, the moth eye structure with silver film developed by our institute can still improve the light penetration and reduce the medial reflection compared with the commercial metal film on the premise of blocking a large amount of thermal radiation. It can be seen that the reflectance of the Ag film on the moth-eye structure are large-area broadband light absorption at the wavelength of infrared region in this study. Our thermal insulation films outperform RAL (AL-608) products offerings in the ultraviolet, while the infrared spectral range is low and sufficiently high in the visible spectral range.

**Table 1:** The transmission and reflectance of two commercially available and the one fabricated in the current study are summarized in different wavelength.

Type	Wavelength			
	400 to 800 nm		850 to 1800 nm	
This study (motheye coating without Ag)	T (%)	92.8	T (%)	91.6
This study (motheye coating with 18 nm Ag)	T (%)	65	T (%)	24
SunMark (K-100SR)	T (%)	56	T (%)	42
RAL (AL-608)	T (%)	26	T (%)	13

## Conclusions

In this study, we develop a cost-effective, efficient approach to enhancing the performance of AR functionality. In the visible spectral range, a sufficiently thick silver layer deposited on AR film offers high transmittance, 72% (Highest value), and in the infrared range, high reflectance (At least 60%). The AR film coated with silver not only performs well in heat insulation, but shows promise in home and vehicle window applications while outperforming existing RAL (AL-608) products.

## Supplementary Information

Supplementary Information.

## Data Availability

All data generated or analysed during this study are included in this published article and its supplementary information files.
