# Supplementary material for: Spectrally selective antireflection of nanoimprint lithography-formed 3D spherical structures on film coated with a silver layer
Source: Sci Rep. 2022 Nov 14;12:19505. doi: 10.1038/s41598-022-23348-w (PMC9663717; doi:10.1038/s41598-022-23348-w)
Supplement: Supplementary file 1 — Supplementary Information. [file 41598_2022_23348_MOESM1_ESM.pdf]

Table S1 the comparison of parameters and reflectivity and transmission based on different metal-based moth-eye structure

ARC Coating Material	*ARC Structure*	*Fabrication Technology*	*R(%)*	*T(%)*	*Wavelength Range(nm)*	*Reference*
Au	Nanocone arrays	Gold vapor deposition	1%	-	450-950	[1]
Au(25nm)	Moth eye structure	plasma-based approach	7.2	48.2	550	[2]
ZnO	Moth eye structure	Aqueous solution method	1.46	-	200-800	[3]
TiO_2	Porous film	Sol-gel based self-assembly and plasma-based approach	-	95	400-900	[4]
Without Ag	Moth-eye structure	Roll-to-Roll	5.2	92.8	400-800	This study
Ag (18nm)	Moth-eye structure	Plasma-enhanced magnetron sputtering	16.4	42.7	400-800	This study

Ref.:

[1] Xu, S.-T.; Hu, F.-T.; Chen, M.; Fan, F.; Chang, S.-J. Broadband Terahertz Polarization Converter and Asymmetric Transmission Based on Coupled Dielectric-Metal Grating. Ann. Der Phys. 529, 1700151. https://doi.org/10.1002/andp.201700151 (2017).

[2] Tsai, H. Y., & Ting, C. J. Optical characteristics of gold film on the moth-eye structure. Current Applied Physics. 12, S156-S159. https://doi.org/10.1016/j.cap.2012.02.049 (2012),

[3] Shin, B.-K.; Lee, T.-I.; Xiong, J.; Hwang, C.; Noh, G.; Cho, J.-H.; Myoung, J.-M. Bottom-up grown ZnO nanorods for an antireflective moth-eye structure on $CuInGaSe_2$ solar cells. Sol. Energy Mater. Sol. Cells95, 2650–2654. https://doi.org/10.1016/j.solmat.2011.05.033 (2011)

[4] Adak, D.; Ghosh, S.; Chakraborty, P.; Srivatsa, K.M.K.; Mondal, A.; Saha, H.; Mukherjee, R.; Bhattacharyya, R. Non lithographic block copolymer directed self-assembled and plasma treated self-cleaning transparent coating for photovoltaic modules and other solar energy devices. Sol. Energy Mater. Sol. Cells 188, 127–139. https://doi.org/10.1016/j.solmat.2018.08.011 (2018).
